# Editorial: Emerging mosquito-borne diseases and novel biocontrol strategies

**DOI:** 10.3389/fcimb.2023.1143165

**Published:** 2023-02-10

**Authors:** Sheng-Qun Deng, Emad I. M. Khater, Ernest Tambo, Duo-Quan Wang

**Affiliations:** ^1^ Department of Pathogen Biology, the Key Laboratory of Microbiology and Parasitology of Anhui Province, the Key Laboratory of Zoonoses of High Institutions in Anhui, School of Basic Medical Sciences, Anhui Medical University, Hefei, China; ^2^ Department of Entomology, Faculty of Science, Ain Sham University, Cairo, Egypt; ^3^ Public Health Pests Laboratory, Municipality of Jeddah Governorate, Jeddah, Saudi Arabia; ^4^ National National Institute of Parasitic Diseases, Chinese Center for Disease Control and Prevention (Chinese Center for Tropical Diseases Research), National Health Commission Key Laboratory of Parasite and Vector Biology, WHO Collaborating Center for Tropical Diseases, National Center for International Research on Tropical Diseases, Shanghai, China

**Keywords:** mosquito-borne disease, biocontrol, malaria, dengue virus (DENV), Zika virus

Mosquito-borne diseases threaten more than 40% of the world’s population and are an increasingly serious global health challenge (Franklinos et al., 2019). A report released by the World Health Organization (WHO) showed that malaria caused 247 million cases and 619,000 deaths in 2021, and there is no significant progress in current malaria control (World Health Organization, 2023a). The global incidence and number of reported epidemic areas of dengue have also grown dramatically (World Health Organization, 2023). Moreover, Zika, a newly emerged mosquito-borne disease associated with neurological complications, has recently caused several large outbreaks involving 89 countries and territories (World Health Organization, 2023b). Furthermore, no efficient vaccines or drugs for diseases such as dengue and Zika are publicly available, and vector control remains largely dependent on traditional insecticide-based strategies (Namias et al., 2021).

Notably, the limitation of the current vector control effect is partly due to the overreliance on chemical control (Fernandes et al., 2018). Chemical insecticides used to be the primary strategy for mosquito control, but insecticide resistance has widely emerged in mosquitoes in recent years (World Health Organization, 2018; Peng et al., 2022). Extensive use of insecticides both in mosquito control and agriculture led to environmental pollution and exerted effects on non-targeted organisms (Deng et al., 2019). Thus, there is a growing need for more sustainable, environmentally friendly, and low-cost vector control strategies that can be implemented on a large scale to harness insecticide-resistant mosquitoes and reduce mosquito-borne disease burden.

Biological control agents are important alternatives or complements to chemical insecticides. Combined with genetic approaches (e.g., transgenesis and paratransgenesis) and other biological rear and release theories, novel approaches, including entomopathogenic fungi (*Metarhizium anisopliae* and *Beauveria bassiana*) (Deng et al., 2019; Peng et al., 2022), symbiotic bacteria (*Wolbachia*) (Turelli et al., 2022), lethal bacteria (*Bacillus thuringiensis*) (Brühl et al., 2020), and the release of sterile male mosquitoes (Wang et al., 2023) or disease-refractory mosquitoes (introducing a pathogen effector gene to replace populations) (Gao et al., 2020; Chen et al., 2023), shed light on a promising future harnessing insecticide resistance. These strategies are sustainable, inexpensive, and safe for humans and create no pollution to the environment. Gene-drive-based technologies have been encouraged to be combined with biological strategies by the World Health Organization Vector Control Advisory Group due to their broad utility in biological strategies and potential to overcome challenges in current vector control (Wang et al., 2021; World Health Organization, 2022). Further epidemiological evidence and field-trial evaluation are needed to support the implementation of these biological measures on a large scale.

This Research Topic, “*Emerging Mosquito-Borne Diseases and Novel Biocontrol Strategies*”, focuses on current and sound research addressing one or more of the abovementioned biocontrol strategies, related genomic surveillance, evolutionary genomics of mosquito species, and insecticide resistance. The Research Topic brings a collection of three original research articles and two reviews. A systematic review and meta-analysis by Wu et al. addressed the impact of COVID-19 non-pharmacological interventions (NPIs) on dengue infection. They searched all qualified articles focusing on NPI efficacy on dengue infection and collected public data on dengue cases to analyze their effects more comprehensively. The study stressed that the changing intensity and scope of internal movement restrictions are more likely to reduce the fundamental level of dengue transmission by reducing the spread of dengue fever among regions in a country, which is conducive to the development of a more comprehensive and sustainable strategy to control dengue fever. Another review by Hou et al. summarized the current development of tetravalent live-attenuated dengue vaccines. CYD-TDV developed by Sanofi Pasteur has been approved, but it is limited to patients who have been infected with dengue fever in the past. The other two candidates for the tetravalent live-attenuated vaccine, TAK-003 of Takeda and TV003 of the National Institute of Allergy and Infectious Diseases, have completed phase III and phase II clinical trials, respectively. They emphasized the specific lessons in the existing research and the challenges that must be overcome in the development of the dengue vaccine, which can effectively protect from all four dengue virus serotypes while causing the fewest side effects. Moreover, Meuren et al. demonstrated that mitochondrial-derived reactive oxygen species (ROS) were a significant inducer of human brain microvascular endothelial cell permeability. In contrast, NADPH oxidase-derived ROS were relevant in producing inflammatory mediators and endothelial activation.

In addition, a study by Qin et al. using the human hepatoma cell model (Huh7), explored the roles of 5’ adenosine monophosphate-activated protein kinase (AMPK), its downstream unc-51-like kinase 1 (ULK1), and mammalian target of rapamycin (mTOR) signaling pathways during the Zika virus infection process. They suggested that Zika virus infection triggers AMPK-mediated lipophagy and that lipid droplet-related lipid metabolism is mainly regulated by the AMPK-ULK1 signaling pathway.

Furthermore, Kimingi et al. used controlled human malaria infection (CHMI) studies in Kenya to further explore the role of anti-*Plasmodium falciparum* variant surface antigen (VSA) antibodies in malaria immunity. The breadth of IgG antibodies against VSAs is related to the protection in CHMI rather than against individual isolate VSAs.

In conclusion, although this special issue does not include enough articles on biological control strategies, it provides new reference materials for researching malaria, dengue, and Zika. The emergence and re-emergence of mosquito-borne diseases deserve our attention, and new biological control methods deserve our in-depth exploration.

## Author contributions

All authors listed have made substantial, direct, and intellectual contributions to the work and approved it for publication.
